# Plasma deoxyuridine as a surrogate marker for toxicity and early clinical response in patients with metastatic colorectal cancer after 5-FU-based therapy in combination with arfolitixorin

**DOI:** 10.1007/s00280-020-04173-2

**Published:** 2020-10-24

**Authors:** Helena Taflin, Elisabeth Odin, Göran Carlsson, Roger Tell, Bengt Gustavsson, Yvonne Wettergren

**Affiliations:** 1 Department of Surgery, The Institute of Clinical Sciences, The Sahlgrenska Academy At University of Gothenburg, Göteborg, Sweden; 2 Isofol Medical AB, Göteborg, Sweden

**Keywords:** Metastatic, 5-Fluorouracil, Folate, Gender, Gastrointestinal toxicity, Haematological toxicity

## Abstract

**Purpose:**

The aim was to explore the correlation between increasing doses of [6R]-5,10-methylenetetrahydrofolate (arfolitixorin) and plasma concentrations of deoxyuridine (dUr) in patients with metastatic colorectal cancer (mCRC), subjected to 5-fluorouracil (5-FU)-based chemotherapy. The aim was further to investigate the possibility to predict toxicity and clinical response during treatment using gender, age, and plasma dUr as explanatory variables.

**Methods:**

Thirty-three patients from the ISO-CC-005 phase I/IIa study, which investigated safety and tolerability of arfolitixorin at four dose levels, were included. Toxicity and clinical response were evaluated after 4 cycles of chemotherapy. Plasma dUr was quantified before (0 h) and 24 h after 5-FU administration at the first (C1) and fourth (C4) cycle using LC–MS/MS. Fit modelling was used to predict toxicity and clinical response.

**Results:**

The dUr levels increased with increasing arfolitixorin dose. Females had higher total and haematological toxicity scores (*p* = 0.0004 and 0.0089, respectively), and needed dose reduction more often than males (*p* = 0.012). Fit modeling showed that gender and the dUr levels at C1-0 h and C4-24 h predicted total toxicity (*p* = 0.0011), whereas dUr C4-0 h alone was associated with gastrointestinal toxicity (*p* = 0.026). Haematological toxicity was predicted by gender and age (*p* = 0.0071). The haematological toxicity score in combination with the dUr levels at C1-24 h and C4-24 h predicted early clinical response (*p* = 0.018).

**Conclusion:**

The dUr level before and during administration of 5-FU and arfolitixorin was predictive for toxicity and early clinical response and could be a potential surrogate marker for thymidylate synthase inhibition in patients with mCRC.

**Trial registration:**

NCT02244632, first posted on ClinicalTrials.gov on September 19, 2014

**Electronic supplementary material:**

The online version of this article (10.1007/s00280-020-04173-2) contains supplementary material, which is available to authorized users.

## Introduction

Colorectal cancer (CRC) is ranked as the third most common cancer worldwide, and in 2018, approximately 1.8 million people were diagnosed with the disease [1]. The base treatment with curable intent for CRC is radical surgery, and for selected rectal tumors, surgery in combination with radiotherapy [2]. Chemotherapy is used in all settings, both neoadjuvant, with the intent of reducing the tumor burden and making the tumor resectable/less advanced; in the adjuvant setting after radical surgery to reduce the risk of recurrence; and in a pure palliative setting with the purpose of prolonging life [3, 4]. Although novel combination treatments have been developed during the last years, 5-fluorouracil (5-FU) plus the folate leucovorin ([6R,S]-5-formyltetrahydrofolate) is still a cornerstone in treatment of CRC [5].

**Fig. 1:**
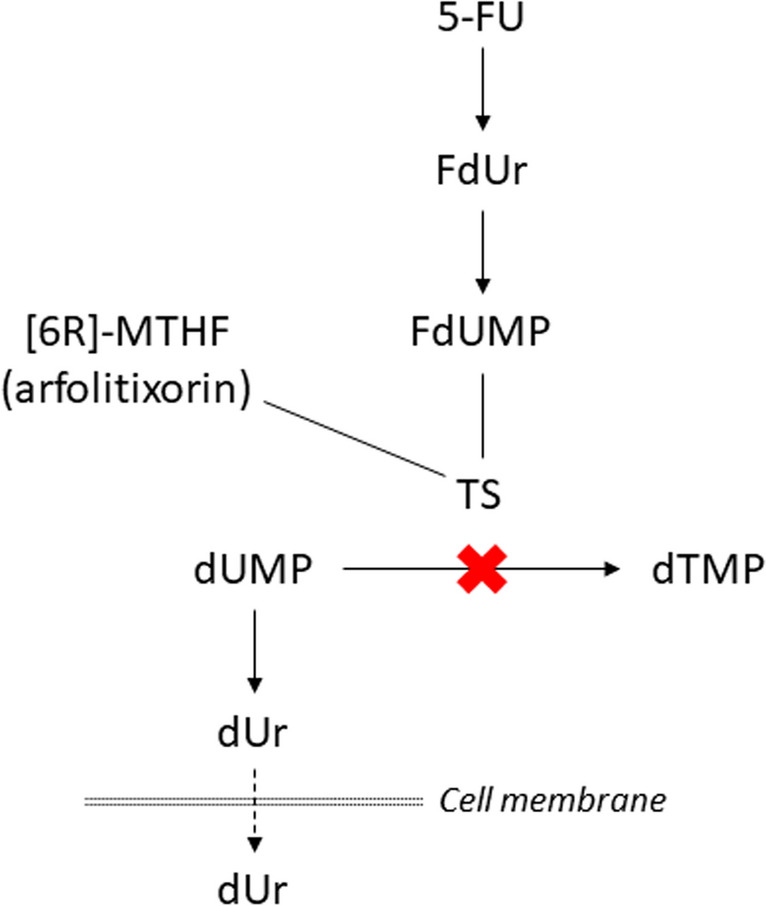
Thymidylate synthase (TS) inhibitory effect on the pathway of thymidine nucleotides. Ternary complex formation with 5-fluorodeoxyuridine monophosphate (FdUMP) blocks the binding of deoxyuridine monophosphate (dUMP) to TS. Dephosphorylation of dUMP to deoxyuridine (dUr) leads to subsequent efflux of dUr into the blood circulation. 5-FU, 5-fluorouracil; dTMP, deoxythymidine monophosphate; FdUr, 5-fluorodeoxyuridine; THF, tetrahydrofolate

5-FU is an analogue of uracil in which the hydrogen at position 5 is replaced by fluorine. Using the same mechanism to enter the cell as uracil, 5-FU is converted into the active metabolite fluorodeoxyuridine monophosphate (FdUMP), which forms an inhibitory ternary complex with one of the key enzymes in DNA synthesis, thymidylate synthase (TS), and [6R]-5,10-methylenetetrahydrofolate ([6R]-MTHF). This results in the inhibition of thymidylate synthesis and impairment of both DNA synthesis and DNA repair (Fig. 1). TS inhibition causes a rise in the intracellular pool of the natural TS substrate deoxyuridine monophosphate (dUMP), leading to increased levels of deoxyuridine (dUr) in tissues and blood [6]. The greatest impact of treatment with 5-FU is on rapidly dividing cells, such as epithelial cells and most importantly, tumor cells [7].

The response rate of colorectal tumors to 5-FU monotherapy is only around 10%. By adding leucovorin (the stable calcium salt of [6R,S]-5-formyltetrahydrofolic acid, i.e., calcium folinate), which is converted to [6R]-MTHF in the liver, the tumor response rate can be improved to 21% [8]. All marketed folates used in established regimes, require intracellular enzymatic conversion to the active metabolite [6R]-MTHF to have a clinical effect.

Previous studies have shown that there is a large inter-individual difference in concentration of leucovorin in colorectal tumor tissue in response to a standardized dose of leucovorin [9]. This difference might be related to different expression levels of genes encoding influx and efflux folate transporters and of those involved in conversion of leucovorin to the active metabolite [6R]-MTHF [10–12]. Gender- and age-associated pharmacokinetic differences have also been reported for folates and most oncologic drugs [13–15]. Female gender and older age are especially associated with severe toxicity during 5-FU-based chemotherapy and may affect clinical response [16, 17].

Arfolitixorin is the hemisulfate salt of [6R]-MTHF which is a stable formulation of the naturally occurring diastereoisomer of [6R]-MTHF [18]. Unlike commonly used folates, [6R]-MTHF does not need to undergo further metabolism and may participate directly in the formation of the FdUMP-TS-MTHF ternary complex. Hence, arfolitixorin is expected to be efficacious in a larger proportion of patients with less inter- and intra-individual variability [19]. Arfolitixorin was previously compared to levo-leucovorin in a blinded, randomized trial including 32 patients scheduled for surgical resection due to colon cancer [18]. The folates were given as a peroperative single intravenous bolus dose of either 60 mg/m^2^ or 200 mg/m^2^. Tumor and mucosa tissue were obtained at surgery after administration of the folates. The results showed that the [6R]-MTHF and THF concentrations were significantly higher after administration of arfolitixorin compared to levo-leucovorin in both tumors and mucosa. These results indicate that there is a higher probability of reaching an optimal level of [6R]-MTHF after arfolitixorin administration.

The aim of the present study was to explore the correlation between increasing doses of arfolitixorin and plasma concentrations of dUr in patients with metastatic CRC (mCRC), subjected to arfolitixorin in combination with chemotherapy. The aim was further to investigate the possibility to predict the risk of toxicity and early clinical response during treatment using gender, age and the plasma dUr level as explanatory variables.

## Materials and methods

### Study design

The patients were all recruited from the ISO-CC-005 (NCT02244632) study, an open-label, multi-center, Phase I/IIa dose cohort trial of patients with mCRC treated with arfolitixorin in combination with a fixed dose of 5-FU, alone, or in combination with a fixed dose of oxaliplatin or irinotecan, and an optional addition of bevacizumab after eight weeks of treatment (Online Resource 1). The main inclusion criteria were mCRC, confirmed by biopsy from either primary tumor or metastatic sites, eligibility for 5-FU-based chemotherapy given as palliative treatment, age ≥ 18 years, World Health Organization (WHO) performance status of 0–2, and a life expectancy exceeding three months. The primary objective of the study was to characterise the safety and tolerability of escalating doses of arfolitixorin in combination with standardized chemotherapy regimens in different treatment lines in terms of toxicity during eight weeks of treatment. Although ISO-CC-005 was an international multicentre study with several recruiting sites in Europe, only one centre (Sahlgrenska University Hospital/Östra, Gothenburg Sweden) was selected to perform pharmacokinetic/pharmacodynamic research.

### Patients

Thirty-six patients were initially recruited for the pharmacokinetic/pharmacodynamic studies. Out of these, two were excluded due to withdrawal of informed consent, and one due to refusal of further treatment resulting in a cohort of 33 patients who received first-line (*n* = 23), second- (*n* = 8), or third-line (*n* = 2) treatment. Nineteen of the patients were male, whereas 14 were female. The median age was 66 years range 38–85). The primary tumors were located in the colon in 25 patients, and in the rectum in 8 patients. Demographic and clinicopathological characteristics of the patients are presented in Online Resource 2.

### Treatment and response evaluation

The palliative treatment consisted of 5-FU and arfolitixorin as single treatment or in combination with irinotecan or oxaliplatin ± bevacizumab for eight weeks (Online Resource 3). Four different doses of arfolitixorin were used: 30, 60, 120 and 240 mg/m^2^ administered two days in a row every two weeks. Treatment efficacy was evaluated by CT scans of the thorax/abdomen according to RECIST1.1 criteria. Clinical response was evaluated as complete response (CR), partial response (PR), stable disease (SD), or progressive disease (PD) after four cycles of chemotherapy.

### Toxicity evaluation

Patients were assessed for toxicity grade after each treatment cycle during the four cycles of chemotherapy, using the National Cancer Institute’s Common Terminology Criteria for Adverse Events (NCI-CTCAE) version 4.0. Toxicities known to be commonly related with given treatment (diarrhea, nausea, vomiting, stomatitis, leucopenia, neutropenia, thrombocytopenia, ocular toxicity, paresthesia, fatigue, and neuropathy) [20, 21] were selected for analysis and the highest toxicity grade during the four treatment cycles was noted. The total toxicity score of a patient was calculated by adding up the toxicity grades (0–4) of each evaluated side effect. Thus, the minimal score would be zero, and the maximal score would be 44. A gastrointestinal toxicity score including diarrhea, nausea, vomiting, and stomatitis was also calculated, as well as a haematological score including leucopenia, neutropenia, and thrombocytopenia. Furthermore, the need of dose reduction during treatment was recorded as yes or no.

### Sample collection

To assess the level of overall TS inhibition, reflected as the change in dUr levels over time, blood samples were collected in pre-chilled heparinized tubes immediately before (0 h), 24 h (24 h), and 48 h (48 h) after administration of the combination treatment on Days 1 and 2 of Cycle 1 (C1) and Cycle 4 (C4). Plasma was immediately isolated from the blood by centrifugation at 1520 g (4 °C, 10 min). The plasma was kept frozen at −80 °C until analysis.

### dUr analysis

A method employing liquid chromatography mass spectrometry (LC–MS/MS) was developed for quantification of plasma dUr using chlorodeoxyuridine (CldUr) as internal standard (IS). It involved precipitation of protein by mixing 60 µl of 1 M trichloroacetic acid (TCA) with 300 µl of plasma. After cooling the mixture on ice for 10 min, 15 µl of 0.5 mM CldUr was added. The mixture was vortexed and centrifuged at 21500xg for 10 min at 8ºC. The sample was then transferred to a 10 kDa molecular weight cut-off membrane filter (Millipore) and centrifuged at 21500 × *g* for 20 min. The solution at the bottom of the centrifugation tube was analyzed on LC–MS/MS. Chromatographic separations were performed on a dC18 Atlantis column. The mobile phases were (A) 5 mM acetic acid (HAc) and (B) 5 mM HAc dissolved in 90% acetonitrile. The gradient profile over time is shown in Online Resource 4 and 5. The analytes were ionized in an electrospray interface (ESI) under negative-ion mode (Online Resource 6).

Linearity was assessed by constructing matrix‐matched calibration curves (*n* = 10) of dUr. Calibration curves were linear over 2–275 pmol dUr. The mean equations from 10 calibration curves were found to be *y* = 0.002435 × −0.00020. The least square regression calibration curve was *R*^2^ = 0.9987 for dUr in plasma. Sensitivity was assessed by evaluating the limit of detection (LOD) and limit of quantification (LOQ) for the method. The LOD and LOQ values were defined as the lowest analyte concentration that could be detected. The LOD for dUr was 0.017 µM and the LOQ was found to be 0.052 µM. Intraday precision was determined by running replicates (*n* = 5) at three levels (low, medium and high) of known dUr concentrations spiked in plasma on the same day. Inter‐assay variability was determined by analyzing low, medium and high dUr concentration samples on 10 separate days. The precision was calculated from the relative standard deviation (RSD %) and ranged from 2.9 to 8.5% whereas the variability over 10 days ranged from 7.6 to 9.8%.

### Statistics

The JMP 13.0/SAS software (SAS Institute Inc. Cary, NC, USA) was used for the statistical analyses. Data were presented as means and standard deviations (SD) or as median values with ranges. Differences between groups were calculated using the Kruskal–Wallis test or the Pearson’s Chi-square test. To compare sets of continuous parameters measured in the same sample (matched pairs), the Pearson correlation coefficient (*r*) was calculated. Fit modelling by effect likelihood ratio tests was used to predict the effect of explanatory variables on toxicity and clinical response during chemotherapy. No correction for multiple testing was done. Values of *p* < 0.05 were considered significant.

## Results

### Toxicity scores versus gender and age

**Fig. 2:**
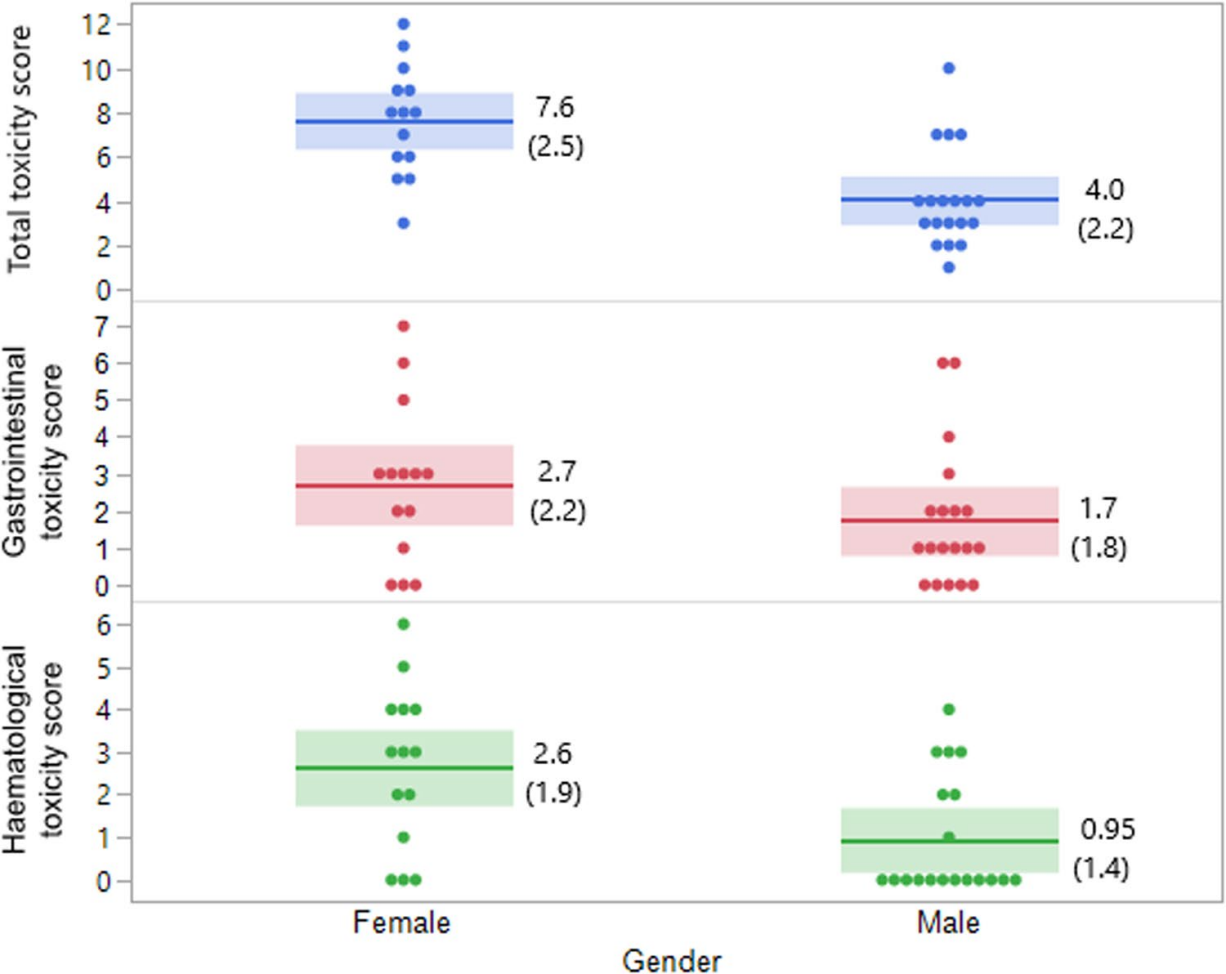
Comparison of the toxicity scores of female and male patients. As shown, females had higher toxicity scores than males. The scores were significantly different for total toxicity (*p* = 0.0004) and haematological toxicity (*p* = 0.0089), but not for gastrointestinal toxicity (*p* = 0.14). Mean values and standard deviations (within parenthesis) are presented to the right of each plot, and confidence intervals are shown as blue- and red-shaded areas. Each dot represents one patient

The mean total, gastrointestinal, and haematological toxicity scores were 5.6 ± 2.9, 2.2 ± 2.0, and 1.7 ± 1.8, respectively. Female patients had higher toxicity scores compared with male patients (Fig. 2). The results also showed that more females than males needed to reduce the dose of chemotherapy during treatment (*p* = 0.013). There was a negative correlation between the gastrointestinal toxicity score and age (*r* = −0.35, *p* = 0.044), however, no significant correlation was seen between total toxicity score or haematological toxicity score and age.

### Clinical response by gender and age

Fifty percent of the female patients had PR, compared to 26.3% of the males. In contrast, more males had SD (52.6%) compared to females (28.6%). However, the number of males and females with PD was equal (21.1% and 21.4%, respectively). The mean age was significantly lower in patients with PD (55.1 ± 11.3 years), compared to patients with SD/PR (66.1 ± 8.4 years, *p* = 0.021).

### Clinical response by toxicity scores

Patients who responded well to treatment (PR) had a higher mean haematological toxicity score compared to patients with stable or progressive disease (Online Resource 7). However, a significant association was not noted between clinical response and the total toxicity score or the gastrointestinal toxicity score.

### Plasma dUr analysis

The mean ± SD concentrations of dUr are presented in Online Resource 8. As shown, there was a large inter-individual variation in dUr plasma levels. There was an increase in dUr levels at C4-0 h and C4-24 h, compared to C1-0 h and C1-24 h. This increase was not seen at 48 h. The dUr levels were higher in females compared to males at all measurements; however, the difference was not significant. No correlation between the dUr levels and age was seen.

**Fig. 3:**
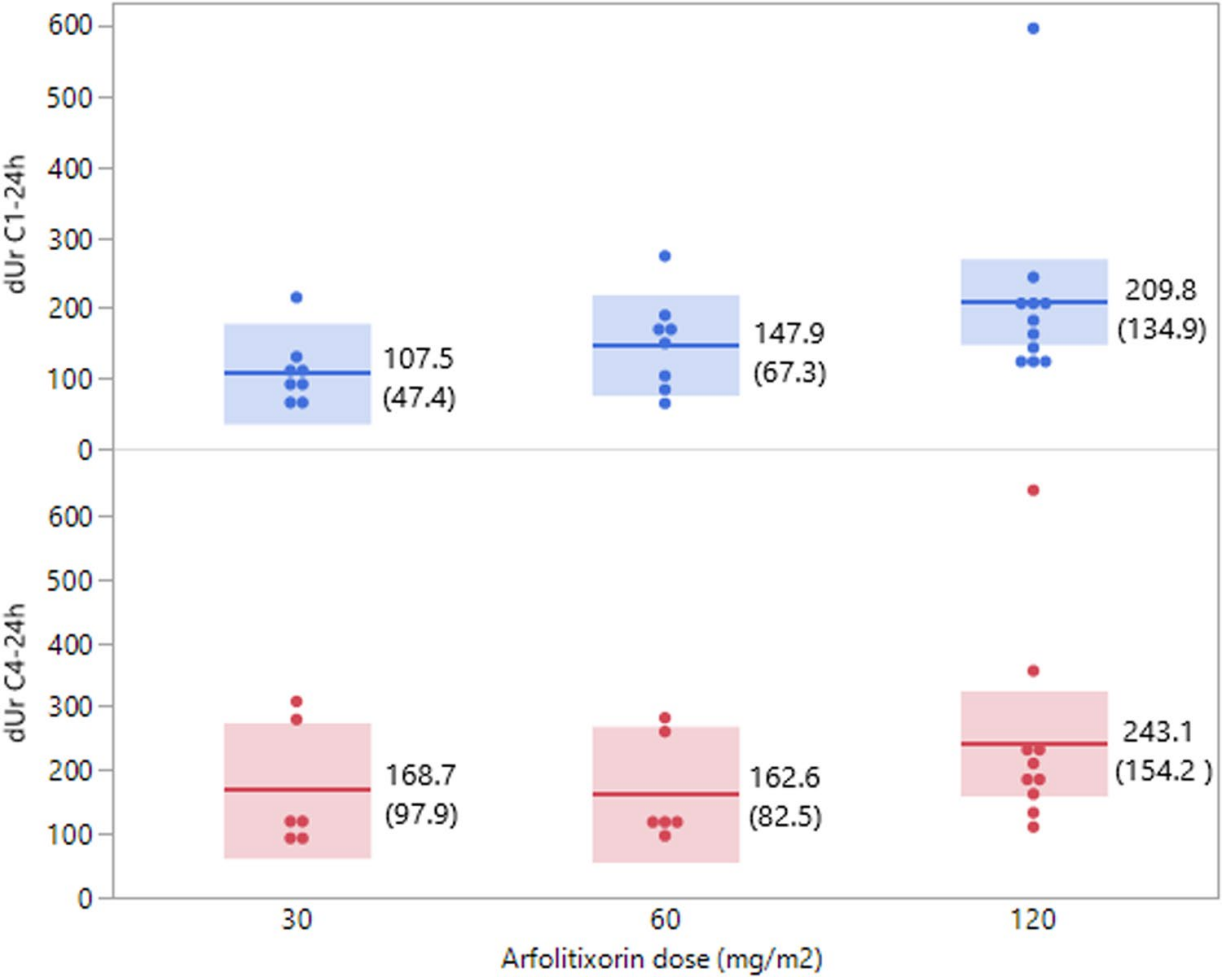
Mean dUr levels at C1-24 h and C4-24 h by arfolitixorin dose. As shown, the dUr C1-24 h levels increased by increasing arfolitixorin dose (*p* = 0.023). At C4-24 h, the plasma dUr level was higher in patients receiving 120, compared to 30 and 60 mg/m^2^ arfolitixorin but the difference did not reach significance (*p* = 0.31). Mean values and standard deviations (within parenthesis) are presented to the right of each plot, and confidence intervals are shown as blue- and red-shaded areas. Each dot represents one patient

The dUr levels at C1 and C4 were analyzed according to given arfolitixorin dose. Since dUr values at C4 were only available for two of the patients receiving 240 mg/m^2^ arfolitixorin, these values were not included in the statistical analysis. The results showed that the plasma dUr levels at C1-24 h increased with increasing dose of arfolitixorin (Fig. 3). As shown in the figure, one patient who received 120 mg/m^2^ arfolitixorin was an outlier, having unusually high dUr levels. When omitting this patient from the statistical analysis, the difference between the groups was still significant (*p* = 0.040). At C4-24 h, the plasma dUr level was higher in patients receiving 120, compared to 30 and 60 mg/m^2^ arfolitixorin. However, the difference did not reach significance.

### Plasma dUr and toxicity

The plasma dUr values were analyzed in patients according to need of dose reduction due to toxicity during chemotherapy (Table 1). As shown, patients who needed dose reduction had significantly higher dUr levels at C1 and C4, both at 0 h and 24 h, compared to patients without need of dose reduction. Fit modelling predicting toxicity during chemotherapy was performed using effect likelihood ratio tests. The gender, age, and the plasma dUr C1 and dUr C4 values at 0 h and 24 h were used as explanatory variables. Before modelling, the total toxicity score (range 1–12) was divided into three groups (≤ 3, > 3 to ≤ 8, and > 8) to obtain categorical values. The results showed that gender and the dUr levels at C1-0 h and C4-24 h were predictive of total toxicity (Table 2). When the gastrointestinal toxicity score (range 0–7) was divided into two groups (≤ 1 and > 1) and modelled, the results showed that dUr C4-0 h was predictive for gastrointestinal toxicity. A high dUr C4-0 h was associated with a higher toxicity score. The mean haematological toxicity score (range 0–6) was divided into two groups (≤ 2, and > 2) and the fit model showed that gender and age were predictive of haematological toxicity. Female gender and higher age were associated with a higher haematological toxicity.

**Table 1:** Association between the dUr levels (pmol/ml) expressed as means ± standard deviations at C1 and C4 and need of dose reduction (no/yes) during chemotherapy

	Dose reduction		*p* value
	No	Yes	
dUr C1-0 h	51.3 ± 14.6 *n* = 22	71.9 ± 37.5 *n* = 11	0.082
dUr C1-24 h	128.9 ± 54.2 *n* = 19	221.9 ± 139.7 *n* = 10	0.012
dUr C4-0 h	68.7 ± 15.0 *n* = 20	100.3 ± 47.0 *n* = 7	0.038
dUr C4-24 h	153.1 ± 60.0 n = 18	328.2 ± 163.5 *n* = 6	0.0030

*dUr*, deoxyuridine; *C1*, treatment cycle 1; *C4*, treatment cycle 4

**Table 2:** Effect likelihood ratio tests predicting the effect of the explanatory variables on toxicity scores and clinical response

	Explanatory variable	*p* value	*p* value whole model
Toxicity score			
Total	Gender	0.0026	0.0011
	dUr C1-0 h	0.0046	
	dUr C4-24 h	0.0071	
Gastrointestinal	dUr C4-0 h	0.026	0.026
Haematological	Gender	0.0091	0.0071
	Age	0.021	
Clinical response	Haematological toxicity score	0.0050	0.018
	dUr C1-24 h	0.038	
	dUr C4-24 h	0.047	

*dUr*, deoxyuridine; *C1*, treatment cycle 1; *C4*, treatment cycle 4

### Plasma dUr and clinical response

The fit model was used to test the possibility to predict clinical response (PR/SD/PD) using gender, age, the plasma dUr C1 and C4 levels at 0 h and 24 h, and the haematological toxicity score as explanatory variables. The results showed that the haematological toxicity score in combination with the dUr levels at C1-24 h and C4-24 h correlated significantly with clinical response (Table 2). A high haematological toxicity score, a low C1-0 h value, and a high C1-24 h value were associated with a better clinical response.

### Changes in plasma dUr in individual patients

**Fig. 4:**
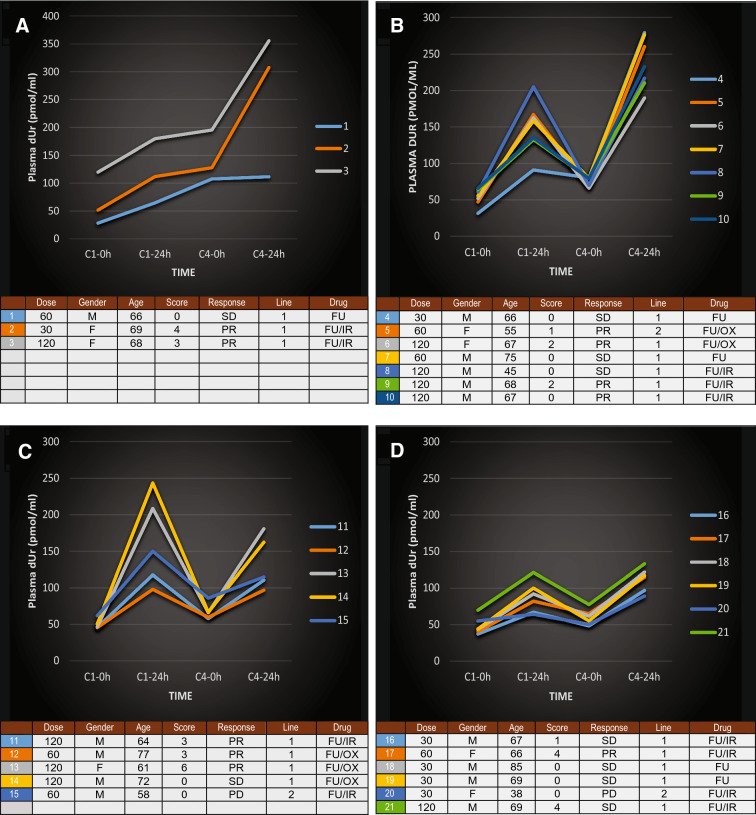
Changes in plasma dUr levels over time in individual patients (*n* = 21) with mCRC, where samples were available at four time points (C1-0 h, C1-24 h, C4-0 h, C4-24 h). Patients were divided into groups based on the dUr alteration patterns. The arfolitixorin dose (mg/m2), gender (M or F), age (years), haematological toxicity score (0–12), clinical response (PR, SD, or PD), treatment line (1 or 2), and cytotoxic drugs [5-FU (FU), 5-FU/oxaliplatin (FU/OX), or 5-FU/irinotecan (FU/IR)] of each patient are presented in tables below the graphs. **a** Three patients increased the dUr levels at each time point. **b** Seven patients had a higher dUr level at C4-24 h compared to C1-24 h. **c** Five patients had a lower dUr level at C4-24 h compared to C1-24 h. **d** Six patients had a higher dUr value at C4-24 h compared to C1-24 h, but the dUr level was low over the entire range. It is striking that 9 out of 10 patients with haematological toxicity had PR, whereas 9 out of 11 patients without haematological toxicity had SD/PD

The plasma dUr levels at C1-0 h, C1-24 h, C4-0 h, and C4-24 h in individual patients (*n* = 21) were plotted into graphs to visualize alterations over time. Patients were divided into four groups based on different alteration patterns (Fig. 4). The gender, age, haematological toxicity score, clinical response, and treatment line of each patient, as well as the arfolitixorin dose and type of cytotoxic drug given, are presented in tables below the graphs. As shown, haematological toxicity was associated with clinical response in almost all of the patients; 9 out of 10 patients with haematological toxicity had PR, whereas 9 out of 11 patients without haematological toxicity had SD/PD.

## Discussion

In the open-label ISO-CC-005 study, 33 patients with mCRC were treated with arfolitixorin in combination with 5-FU, alone, or in combination with a fixed dose of oxaliplatin or irinotecan ± bevacizumab. The study was designed as a phase I/IIa dose limiting toxicity study; thus, the number of patients included in each group was limited. As the small sample size may have affected the results of the statistical analysis, the data need to be treated with caution. A strength of the study was that all patients were treated in a standardized manner by the same medical team at a single center. Another strength was that each patient represented their own control in the pharmacokinetic part of the studies, since the dUr values at baseline were compared with dUr values at different time points during treatment.

Several studies on CRC have shown that females experience a higher degree of toxicity than males [16, 22–25]. For example, a meta-analysis of the toxicity profiles of five CRC trials including more than 2,400 patients showed that females receiving 5-FU-based chemotherapy in a 5-day bolus schedule experienced more frequent and more severe toxicity than males [25]. The gender differences were consistent across treatment cycles. In another large study of CRC patients from four trials, 5-FU toxicity was found to be more extensive in females than in males in terms of maximum toxicity grade and number of different toxicity types. This gender difference persisted across a range of treatment regimens, patient characteristics, and cancer trial settings [24]. Haematological toxicity in particular, has been reported to be higher in females compared to males in some studies [24, 26]. This may be because haematological toxicity variables are measured objectively, which means that they are not subjected to any potential reporting bias. In addition to female gender, high age has been found to predict for increased grade 3/4 leucopenia in patients receiving modulated 5-FU [26].

In the present study, fewer female than male patients (41% vs 59%) participated, thus, the numbers of toxicity-related events were expected to be lower compared to cohorts with an equal gender distribution. Even so, and in line with previous reports, the haematological toxicity score was significantly higher among females than males. As reviewed by Schmetzer and Flörcken [15], one of the reasons for the toxicity-related gender differences is gender-biased expression of metabolic enzymes and transporters in liver and kidney. This may lead to different pharmacokinetics, as has been described for most common anti-cancer drugs. Interestingly, both 5-FU and folates have slower clearance rates in females compared to males, which may explain a part of the increased toxicity [13, 14].

Furthermore, the results of the present study showed that the haematological toxicity score was significantly associated with clinical response. Since the female patients experienced greater haematological toxicity, it was not surprising that they also had a higher rate of PR compared to the male patients (50% vs 26%). Clear gender-dependent differences in response rates correlating with the risk of toxicity during chemotherapy have been reported previously. For instance, Abola et al. found that across 99 randomized oncology trials, treatments with greater all-grade toxicity compared with controls were associated with greater progression-free survival [17]. As is the case for toxicity, response rates often correlate with pharmacokinetics; however, non-pharmacokinetics may also be involved. For instance, it has been suggested that females have lower expression of dihydropyrimidine dehydrogenase (DPD), i.e., the rate-limiting catabolic enzyme for 5-FU, in their colorectal carcinomas which make them more sensitive to 5-FU [27].

As haematologic toxicity is one of the main causes of treatment discontinuation, it is important that the toxicity level reached is not too high, but high enough to achieve a good clinical response. By analyzing predictive markers like dUr in addition to haematological variables, it may be easier to predict the level of toxicity during treatment. When the plasma dUr levels were analyzed in the present study, a large inter-individual variation was found both before and during chemotherapy. One reason for the variation might be that the initial tissue level of dUMP differed among patients. It is known that 5-FU treatment causes increased tissue dUMP levels [28], but if a patient’s initial dUMP concentration is low, the treatment may not lead to a detectable increase in plasma dUr. Similarly, treatment with folate in patients who have an initial tissue folate deficiency may not lead to TS inhibition and an increase in plasma dUr, as there will be a competition for folates between different cellular pathways [29]. Furthermore, in patients with high DPD activity, low FdUMP levels will be generated leading to less TS inhibition and lower levels of plasma dUr.

The advantage of using plasma dUr as a surrogate marker is the possibility to follow individual alterations reflecting TS inhibition over time. This has been tested previously in a few studies. For instance, elevations in dUr levels were found during treatment of patients with CRC using 5-FU or the antifolates raltitrexed and ZD9331 [6]. Significant dose-related increases in plasma dUr were also found in patients having a range of different malignancies, including CRC, and who were treated with ZD9331 [30]. In the present study, the mean dUr levels at C1-24 h increased significantly with increasing arfolitixorin dose, and at C4-24 h, the dUr level was highest in patients who received the highest dose of arfolitixorin. The increase in dUr might reflect a positive effect of arfolitixorin on TS inhibition, which should be associated with a higher risk of toxicity and possibly a better clinical response. In fact, the fit model tests showed that both the dUr C4-24 h and the dUr C1-0 h variables in combination with gender were associated with total toxicity. Gastrointestinal toxicity was associated with the dUr level before treatment at cycle 4, thus, the dUr C4-0 h variable could be a predictive marker for gastrointestinal toxicity during 5-FU treatment. The strongest markers for haematological toxicity, however, were gender and age.

The study was performed over a limited study period; only four cycles of treatment were given, which might have had an impact on response evaluation. Even so, the evaluation after the four-cycle treatment showed very promising results with 78% of the patients having partial response or stable disease. The results further showed that haematological toxicity in combination with the dUr levels at C1-24 h and C4-24 h correlated significantly with clinical response. A high dUr C4-24 h value was associated with a better clinical response, even in patients with second-line treatment. This indicates that the change in dUr levels over time may predict clinical outcome, and that it is possible to use dUr as an early prognostic marker for TS inhibition, easily obtained through a blood sample.

Although most of the patients showed an increase in plasma dUr at C4-24 h compared to C1-24 h, some actually had a lower level. This may be due to inter-individual or gender differences in the transport or metabolism of folates and 5-FU or, as mentioned, the initial concentration of dUMP and folates in tissues. Furthermore, polymorphisms in the TS gene lead to variation in its expression between individuals, which can affect plasma dUr levels as has been shown previously [19, 31, 32]. It is also possible that some patients developed resistance to 5-FU through increased TS gene expression before or during treatment [33]. In future studies, it will be of importance to study if patients who do not increase their plasma dUr levels in response to treatment will benefit from modulation of 5-FU by optimization of the arfolitixorin dose. In this regard, it will also be of value to analyze the plasma 5-FU levels during treatment to determine the 5-FU area under the curve (AUC). A positive correlation between 5-FU AUC and toxicity, as well as clinical response, has been reported recently [34]. A positive correlation between 5-FU AUC and haematological toxicity in particular has also been observed [35]. By monitoring the 5-FU AUC, it might be possible to predict the optimal 5-FU dose of each patient.

Since the ISO-CC-005 study was designed as a phase I/IIa study with focus on safety, the inclusion criteria of the patients were broad, allowing for patients with WHO performance status 0–2, and a life expectancy of at least three months to be included. Thus, some of the patients were frail, which might have influenced the severity of toxicity as well as the clinical response. Also of importance is the fact that a few patients received 5-FU as bolus injection, whereas other received the drug as a combination of bolus injection and continuous infusion*.* The different scheduling of 5-FU might have affected the specificity for TS and duration of TS inhibition, which in turn could have influenced toxicity and response. However, as mentioned, gender-related differences regarding 5-FU-related toxicity seem to be persistent across treatment regimens and different cancer trial settings [24], which strengthen the results.

## Conclusion

Finding markers that reflect TS inhibition during 5-FU-based treatment is essential to identify patients who are at risk of developing severe toxicity and who responds to treatment. Previous studies have suggested that dUr might be a useful surrogate marker of TS inhibition. The present study showed the plasma dUr concentration before and during administration of 5-FU-based chemotherapy modulated by arfolitixorin was significantly associated with both toxicity and early clinical response. Based on these data, we conclude that dUr is a potential surrogate marker for TS inhibition, and that modulation of TS activity might be possible by individualizing the dosage of arfolitixorin. Analysis of plasma dUr as well as other putatively informative 5-FU-related markers will be valuable to improve individualization of chemotherapy. Studies of additional, more homogenous cohorts of patients with CRC are presently ongoing, where separate prediction models will be evaluated for female and male patients. These models will hopefully be useful for development of gender-adapted and individual therapies.

## Availability of data and material

The datasets used and/or analyzed during the current study are available from the corresponding author on reasonable request.

## Electronic supplementary material

Below is the link to the electronic supplementary material.

Online Resource 1 ISO-CC-005 (NCT02244632) was a multi-center, phase I/IIa study on mCRC patients eligible for 5-FU/folate therapy alone or in combination with irinotecan or oxaliplatin ± bevacizumab. The primary endpoints of the study were safety and tolerability. Additional key inclusion criteria were WHO 0–2, with an estimated survival of greater than three months. The folate used in the study, arfolitixorin, was tested at four different doses: 30, 60, 120 and 240 mg/m2, together with 5-FU given both as a bolus and as an infusion regimen. The treatment was administered every two weeks (PNG 111 kb)

Supplementary file2 (DOCX 12 kb)

Supplementary file3 (DOCX 13 kb)

Supplementary file4 (DOCX 12 kb)

Online Resource 5 A typical chromatogram showing peaks and retention times for deoxyuridine (dUr, upper panel) and the internal standard chlorodeoxyuridine (CldUr, lower panel) in a patient plasma sample 24h after 5-FU treatment (TIF 39 kb)

Supplementary file6 (DOCX 12 kb)

Online Resource 7 Mean toxicity scores by clinical response (PR, SD, PD). The haematological toxicity score was significantly higher in patients who responded to treatment (p = 0.047). In contrast, the scores for total toxicity and gastrointestinal toxicity were not significantly associated with clinical response (p = 0.22 and p = 0.40, respectively). Mean values and standard deviations (within parenthesis) are presented to the right of each plot, and confidence intervals are shown as blue-, red-, and green-shaded areas. Each dot represents one patient (PNG 24 kb)

Supplementary file8 (DOCX 12 kb)
