# Supplementary figures and images for: Plasma deoxyuridine as a surrogate marker for toxicity and early clinical response in patients with metastatic colorectal cancer after 5-FU-based therapy in combination with arfolitixorin
Source: Cancer Chemother Pharmacol. 2020 Oct 24;87(1):31–41. doi: 10.1007/s00280-020-04173-2 (PMC7801297; doi:10.1007/s00280-020-04173-2)

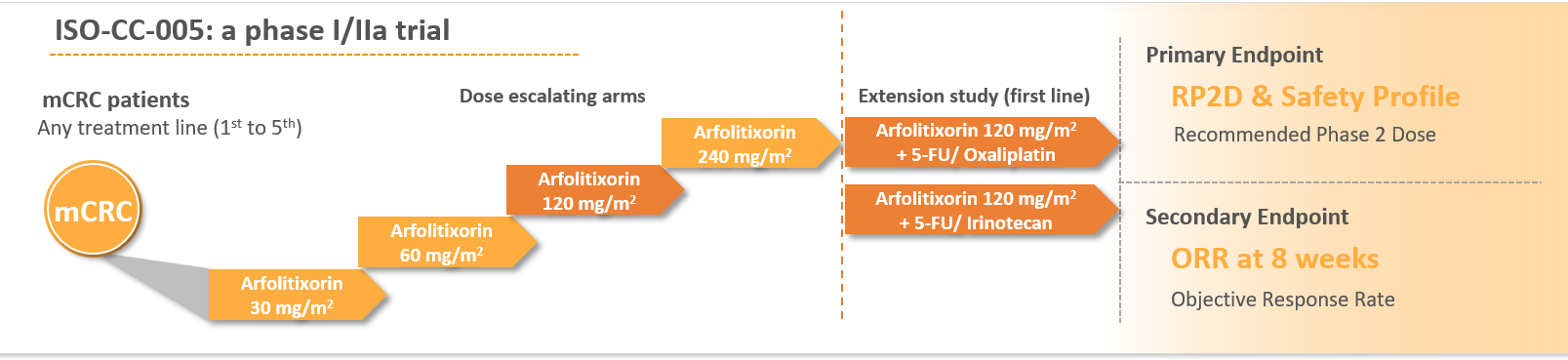

Supplement: Supplementary file 1 — Online Resource 1 ISO-CC-005 (NCT02244632) was a multi-center, phase I/IIa study on mCRC patients eligible for 5-FU/folate therapy alone or in combination with irinotecan or oxaliplatin ± bevacizumab. The primary endpoints of the study were safety and tolerability. Additional key inclusion criteria were WHO 0–2, with an estimated survival of greater than three months. The folate used in the study, arfolitixorin, was tested at four different doses: 30, 60, 120 and 240 mg/m2, together with 5-FU given both as a bolus and as an infusion regimen. The treatment was administered every two weeks (PNG 111 kb) [file 280_2020_4173_MOESM1_ESM.png]

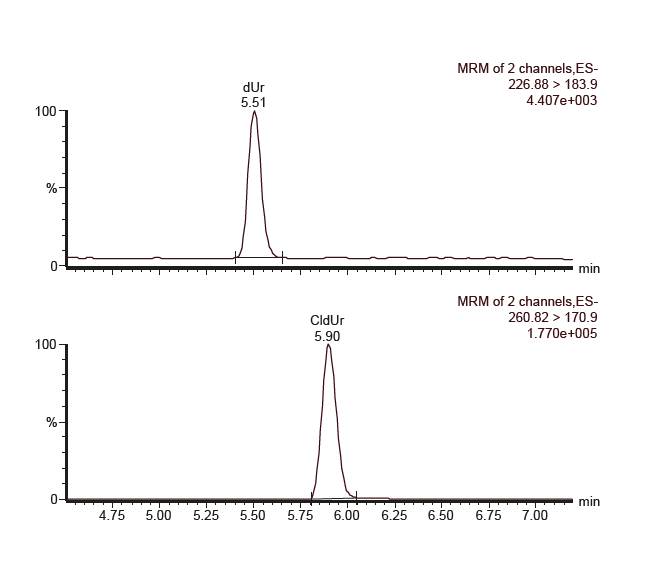

Supplement: Supplementary file 5 — Online Resource 5 A typical chromatogram showing peaks and retention times for deoxyuridine (dUr, upper panel) and the internal standard chlorodeoxyuridine (CldUr, lower panel) in a patient plasma sample 24h after 5-FU treatment (TIF 39 kb) [file 280_2020_4173_MOESM5_ESM.tif]

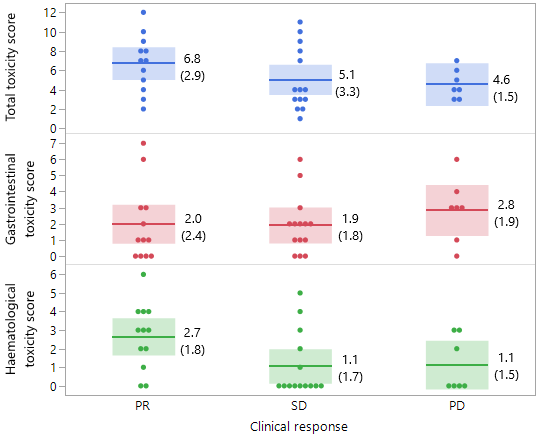

Supplement: Supplementary file 7 — Online Resource 7 Mean toxicity scores by clinical response (PR, SD, PD). The haematological toxicity score was significantly higher in patients who responded to treatment (p = 0.047). In contrast, the scores for total toxicity and gastrointestinal toxicity were not significantly associated with clinical response (p = 0.22 and p = 0.40, respectively). Mean values and standard deviations (within parenthesis) are presented to the right of each plot, and confidence intervals are shown as blue-, red-, and green-shaded areas. Each dot represents one patient (PNG 24 kb) [file 280_2020_4173_MOESM7_ESM.png]
