# Supplementary material for: Plasma deoxyuridine as a surrogate marker for toxicity and early clinical response in patients with metastatic colorectal cancer after 5-FU-based therapy in combination with arfolitixorin
Source: Cancer Chemother Pharmacol. 2020 Oct 24;87(1):31–41. doi: 10.1007/s00280-020-04173-2 (PMC7801297; doi:10.1007/s00280-020-04173-2)
Supplement: Supplementary file 2 — Supplementary file2 (DOCX 12 kb) [file 280_2020_4173_MOESM2_ESM.docx]

| **Online Resource 2** Patient and tumor characteristics | |
| --- | --- |
| Age, years, median (range) | 66 (38-85) |
| Sex, n (%) |  |
| *Male* | 19 (57.6) |
| *Female* | 14 (42.4) |
| Tumor location, n (%) |  |
| *Colon* | 25 (75.8) |
| *Rectum* | 8 (24.2) |
| Treatment line, n (%) |  |
| *First* | 23 (69.7) |
| *Second* | 8 (24.2) |
| *Third* | 2 (6.1) |
| Clinical response, n (%) |  |
| *PR* | 12 (36.4) |
| *SD* | 14 (42.4) |
| *PD* | 7 (21.2) |
| WHO performance status |  |
| *0* | 26 (78.8) |
| *1* | 5 (15.1) |
| *2* | 2 (6.1) |
| PR, partial response; SD, stable disease; PD, progressive disease | |
