# Supplementary material for: Plasma deoxyuridine as a surrogate marker for toxicity and early clinical response in patients with metastatic colorectal cancer after 5-FU-based therapy in combination with arfolitixorin
Source: Cancer Chemother Pharmacol. 2020 Oct 24;87(1):31–41. doi: 10.1007/s00280-020-04173-2 (PMC7801297; doi:10.1007/s00280-020-04173-2)
Supplement: Supplementary file 3 — Supplementary file3 (DOCX 13 kb) [file 280_2020_4173_MOESM3_ESM.docx]

| Online Resource 3 Number of patients in each treatment group | | | | | | |
| --- | --- | --- | --- | --- | --- | --- |
| Number of patients | Arfolitixorin (mg/m^2^) | Chemotherapy/antibody | | | | |
|  |  | 5-FU bolus | 5-FU infusion | Oxaliplatin | Irinotecan | Bevacizumab |
| 3 | 30 | x |  |  |  |  |
| 4 | 30 | x |  |  | x |  |
| 1 | 30 | x |  | x |  |  |
| 2 | 60 | x |  |  |  |  |
| 2 | 60 | x |  |  | x |  |
| 1 | 60 | x | x |  | x | x |
| 4 | 60 | x |  | x |  |  |
| 1 | 60 | x | x | x |  |  |
| 7 | 120 | x | x |  | x |  |
| 4 | 120 | x | x | x |  |  |
| 2 | 240 | x |  |  |  |  |
| 2 | 240 | x | x | x |  |  |
|  | | | | | | |
