# Supplementary material for: Plasma deoxyuridine as a surrogate marker for toxicity and early clinical response in patients with metastatic colorectal cancer after 5-FU-based therapy in combination with arfolitixorin
Source: Cancer Chemother Pharmacol. 2020 Oct 24;87(1):31–41. doi: 10.1007/s00280-020-04173-2 (PMC7801297; doi:10.1007/s00280-020-04173-2)
Supplement: Supplementary file 4 — Supplementary file4 (DOCX 12 kb) [file 280_2020_4173_MOESM4_ESM.docx]

| **Online Resource 4**  Gradient profile | | |
| --- | --- | --- |
| Time | Mobile phase | |
| (min) | A | B |
| 0 | 100 | 0 |
| 1 | 95 | 5 |
| 2 | 92 | 8 |
| 3 | 30 | 70 |
| 4 | 30 | 70 |
| 4.4 | 10 | 90 |
| 5 | 10 | 90 |
| 6 | 0 | 100 |
| 6.5 | 100 | 0 |
| 10 | 100 | 0 |
