# Supplementary material for: Plasma deoxyuridine as a surrogate marker for toxicity and early clinical response in patients with metastatic colorectal cancer after 5-FU-based therapy in combination with arfolitixorin
Source: Cancer Chemother Pharmacol. 2020 Oct 24;87(1):31–41. doi: 10.1007/s00280-020-04173-2 (PMC7801297; doi:10.1007/s00280-020-04173-2)
Supplement: Supplementary file 6 — Supplementary file6 (DOCX 12 kb) [file 280_2020_4173_MOESM6_ESM.docx]

| **Online resource 6** List of compounds and MS parameters | | | | | |
| --- | --- | --- | --- | --- | --- |
| Compound | ESI | Precursor Ion  (m/z) | Product Ion  (m/z) | Cone Voltage (V) | Collision Energy (eV) |
| dUr | - | 227 | 184 | 27 | 11 |
| CldUr | - | 261 | 171 | 29 | 15 |
| dUr, deoxyuridine; CldUr, chlorodeoxyuridine; ESI, electrospray interface | | | | | |
