# Supplementary material for: Plasma deoxyuridine as a surrogate marker for toxicity and early clinical response in patients with metastatic colorectal cancer after 5-FU-based therapy in combination with arfolitixorin
Source: Cancer Chemother Pharmacol. 2020 Oct 24;87(1):31–41. doi: 10.1007/s00280-020-04173-2 (PMC7801297; doi:10.1007/s00280-020-04173-2)
Supplement: Supplementary file 8 — Supplementary file8 (DOCX 12 kb) [file 280_2020_4173_MOESM8_ESM.docx]

| **Online Resource 8** Plasma dUr concentrations (pmol/ml) expressed as means ± standard deviations | | | |
| --- | --- | --- | --- |
|  | 0h | 24h | 48h |
| dUr C1 | 58.2±26.0  n = 33 | 161.0±100.9  n = 29 | 151.0±21.1  n = 4^a^ |
| dUr C4 | 76.9±29.5  n = 27 | 196.9±120.3  n = 24^b^ | 146.9±36.4  n = 2^a^ |
| ^a^Samples were only obtained at 0h and 48h.  ^b^The C4-24h sample was missing from one patient.  dUr, deoxyuridine; C1, treatment cycle 1; C4, treatment cycle 4 | | | |
